# Effect of Colonization of *Trichoderma harzianum* on Growth Development and CBD Content of Hemp (*Cannabis sativa* L.)

**DOI:** 10.3390/microorganisms9030518

**Published:** 2021-03-03

**Authors:** Ioanna Kakabouki, Alexandros Tataridas, Antonios Mavroeidis, Angeliki Kousta, Stella Karydogianni, Charikleia Zisi, Varvara Kouneli, Artemis Konstantinou, Antigolena Folina, Aristidis Konstantas, Panayiota Papastylianou

**Affiliations:** Laboratory of Agronomy, Department of Crop Science, Agricultural University of Athens, 11855 Athens, Greece; a.tataridas@gmail.com (A.T.); antoniosmauroeidis@gmail.com (A.M.); aggelikh.kousta@gmail.com (A.K.); stella.karidogianni@hotmail.com (S.K.); xarikleiazisi@gmail.com (C.Z.); kounelivarvara@gmail.com (V.K.); artemiskonsta24@gmail.com (A.K.); folinanti@gmail.com (A.F.); konar1979@yahoo.gr (A.K.); ppapastyl@aua.gr (P.P.)

**Keywords:** industrial hemp, *Cannabis sativa* L., *Trichoderma harzianum*, CBD, agronomic characteristics, PGPM

## Abstract

*Trichoderma harzianum,* as a natural endophytic biocontrol agent, can ameliorate plant development, nutrient uptake, and resistance to biotic and abiotic stresses. This study aimed to investigate the effect of Trichoderma harzianum inoculation on agronomical and quality characteristics of two monoecious hemp (Cannabis sativa L.) varieties, Fedora 17 and Felina. A greenhouse pot experiment was conducted in a completely randomized design of two treatments of Trichoderma harzianum *with a low and high dose of the fungus (T1 and T2)*. The significance of differences between treatments was estimated by using a Fisher’s test with a significance level *p* = 0.05. The root density of both varieties was significantly affected by treatments, and higher values were recorded in Fedora 17 (2.32 mm cm^−3^). The Arbuscular Mycorrhizal Fungi (AMF) colonization of the root system and the soil emission of CO_2_ were higher after the inoculation of Trichoderma harzianum. The highest values of plant height and dry weight were noticed for T2, especially in variety Felina. Trichoderma harzianum positively influenced characteristics of inflorescences such as their number, fresh weight moisture, and compactness in both varieties, while the dry weight, length, and dry yield of inflorescences were not improved. Finally, the fertigation of *Trichoderma harzianum* in hemp plants was beneficial by increasing the cannabidiol (CBD) content, especially in T2 treatment (4 × 10^12^ CFU kg^−1^).

## 1. Introduction

Industrial hemp (Cannabis sativa L.), which is one of the oldest crops used by humans, has been cultivated for centuries around the world [1]. The significance of hemp derives from its versatility to produce several products with multiple applications such as textile fibers, food, construction materials, and medicines [2,3]. Despite its importance, the cultivation led to a decline during the twentieth century due to the increased use of synthetic fibers and other raw materials and mainly the forbiddance of hemp cultivation as an illicit drug crop owing to the content of psychotropic substance Δ9-tetrahydrocannabinol (THC) [4,5,6].

In the last decades, the crop seems to have regained ground due to the segregation of hemp varieties from psychotropics. Several European Union (EU) countries have authorized the cultivation of hemp varieties that contain THC lower than 0.2% in EC regulation [7,8]. Nowadays, hemp constitutes an area of interest for plenty of researchers and industrial businesses [9]. It is vastly used in more than 25,000 products occupying a prominent position in the global market [10]. More specifically, hemp is used in the textile industry as a green substitute for synthetic fibers, while it is also an important component of other sectors such as food and personal care [11].

Besides the aforementioned uses of hemp, there is currently a rising trend for medicinal hemp compounds in the pharmaceutical industry [12]. Since consumers tend to affiliate healthier habits and prefer medicines produced by natural compounds, the demand for medicinal properties of hemp became even greater. Hemp contains hundreds of secondary metabolites such as terpenes, flavonoids, and cannabinoids [13]. These metabolites are mostly contained in the inflorescences of hemp and are extractable [14]. The most well-known cannabinoids are cannabidiol (CBD), the non-psychoactive section that implies the medicinal properties, and the tetrahydrocannabinol (THC), the primordial psychoactive chemical compound. The suitability of hemp, as a medical compound, is determined by the ratio of CBD to THC [15]. Hemp varieties that are characterized by a great CBD concentration and high ratio of CBD to THC are considered appropriate for the pharmaceutical industry.

Restrictions around the cultivation of this crop resulted in a scientific gap; consequently, there is a deficiency of agronomical information about hemp cultivation, including genotype choice and cultivation practices [16,17,18]. In several European countries, the selection of monoecious dual-purpose varieties over dioecious is a common practice to accomplish mutual fiber and seed production [19]. According to the literature, improved varieties are suitable for multiple exploitations of the crop [20,21], as they form high inflorescences, secure the maximization of CBD production, and have low THC content [22]. However, the adaptability of different hemp varieties in various environments is still an open research field.

From an agronomical point of view, hemp is a high-yield crop with low inputs [6]. Nevertheless, experimental results indicate that hemp demands special attention to nutritional requirements, mainly applied nitrogen (N). Besides, increased nitrogen supply has a positive effect on the agronomical characteristics of hemp, increasing the growth development, plant height, and dry biomass [23]. Several studies mentioned that high doses of N positively affect inflorescence indices [2] and cannabinoid content [24]. While the supply of nitrogen fertilizers is necessary for the optimization of agronomical and quality features of hemp, it is necessary to improve the sustainability of the crop.

In terms of intensive hemp cultivation, constant application of inorganic fertilizers might burden the agroecosystem and harm the environment [25]. Application of plant growth-promoting microorganisms (PGPM) constitutes a specific prospect to lessen the environmental impact and the financial costs of the massive use of inorganic fertilizers [26]. PGPM affects plant nutrition and development via significant processes such as nitrogen stabilization, dissolution of soluble minerals, and release of compounds such as vitamins, enzymes, and phytohormones [27]. Several studies mentioned as an integrated strategy the combination of low rates of inorganic fertilizers and PGPM inoculants, which achieve equivalent results with the high rate of inorganic fertilizers [28,29].

Inoculations with fungus seem to play a crucial role in plant growth, yielding, and improvement of soil fertility compared to many PGPM [30]. Fungal species of the genus *Trichoderma* are free-living fungi used in agriculture for their biocontrol abilities [31,32]. These fungi can resist biotic stresses via mycoparasitism and competition with various phytopathogens [33,34]. This biocontrol agent is observed in many crops such as peanut, tomato, and Chinese cabbage [35,36,37]. Furthermore, some strains of *Trichoderma harzianum* can affect plant growth and nutrient uptake, as well as their defense against abiotic danger [38]. Researchers reported the capacity of *Trichoderma harzianum* to increase the availability of specific nutrition elements in soil, such as nitrogen and phosphorus [39]. As a result, the root system was further developed through the formulation of more branches and root tips as well as the aboveground part of the plant [40]. Moreover, increased root weight in wheat, root length in beans, and higher biomass production of tomatoes have been reported [39,41,42]. After the inoculation with *Trichoderma harzianum*, higher yields have also been recorded in potato, lettuce, and bell pepper plants [40,43,44]. Treatment with *Trichoderma* strains also improved the nutritional quality of end-products [45].

Although there is available literature about the colonization of *Trichoderma* strains on many cultivated plant species, there is a shortage of the effect of these fungi on hemp crops [46]. Therefore, the ultimate goal of this study is to shed light on the effect of *Trichoderma harzianum* on the root growth, vegetative development, and quality features of two monoecious hemp varieties.

## 2. Materials and Methods

A greenhouse pot experiment was conducted in spring 2019 in the Laboratory of Agronomy of Agricultural University of Athens (Southern Greece, latitude: 37°58′ N, longitude: 23°32′ E, altitude 30 m above sea level) to evaluate the effect of *Trichoderma harzianum* on the agronomic and quality traits of two monoecious hemp varieties. Seedlings of *Cannabis sativa* varieties ‘Fedora 17’ and ‘Felina’ were planted in 12 L pots filled with soil and compost (8 L compost per pot). The soil used was clay loam with 29.3% clay, 33.8% silt, and 36.9% sand. The physicochemical properties of soil and compost are presented in Table 1. *Trichoderma harzianum* was the commercial product Trianum-P by Koppert Biological Systems. This product contains water-dispersible granules of 1% *w/w Trichoderma harzianum* strain T-22 with 1 × 10^12^ colony forming units (CFU) kg^−1^. The experiment was set up in a completely randomized design with two treatments of *Trichoderma harzianum*. The split-application was carried out through fertigation 10 and 30 days after sowing (DAS). Treatments and doses applied are presented in Table 2. For the preparation of the suspensions of *Trichoderma harzianum*, the granules were originally dissolved into the water at a ratio of 1:5 (*w*/*v*) (for each g of Trianum-P 5 mL of water were required). These initial suspensions were then diluted, as the water was added to them until their final volume reached 50 L. Treatments were then applied to cannabis plants via fertigation.

Measurements included various assessments of agronomic and quality characteristics of ‘Fedora 17’ and ‘Felina’ plants. All measurements were conducted at the end of the growing season, at 90 DAS, which is considered the optimum stage to evaluate CBD. Plant height, dry weight per plant, the number of buds per plant, bud moisture, and bud fresh and dry weights were estimated based on 4 plants per treatment and per cannabis variety.

Root density was estimated by collecting 4 plants per treatment at 0–35 cm layer by a cylindrical auger (10 cm diameter and 25 cm length). Roots were washed over a 5 mm mesh sieve using a formalin/acetic acid/alcohol (FAA) staining solution. A high-resolution Delta-T Scan version 2.04 (Delta Devices Ltd., Burwell, Cambridge, UK) was used to determine root density (mm cm^−3^) [47,48]. The grid line section method at 30–40 × magnification was utilized to determine microscopically the colonization of the root system by Arbuscular Mycorrhizal Fungi (AMF) [49]. Basal soil respiration (CO2-C) was determined using the titration method [50].

CBD (%) content was estimated in GemmaCert device machine (GemmaCert Ltd., Ra’anana, Israel). Yield per plant (g) for each variety was measured by ten inflorescences from each treatment. Fresh inflorescences were dried for 48 h at 60 °C. The compact bud index was calculated with the formula below.

(1) $$\begin{array}{l} \mathrm{bud}\;\mathrm{compact}\;\mathrm{index}= \end{array}\frac{\begin{array}{l} \mathrm{bud}\;\mathrm{dry}\;\mathrm{weight}\;\left(g\right) \end{array}}{\begin{array}{l} \mathrm{bud}\;\mathrm{length}\;\left(\mathrm{cm}\right) \end{array}}$$

Analysis of variance was carried out using the STATISTICA v10 (StatSoft, Inc., Tulsa, OK, USA, 2011) logistic package as a completely randomized design. Significant differences between treatments were compared by using Fisher’s least significant difference (LSD) test where probabilities equal to or less than 0.05 and were considered significant. Correlation coefficients and linear regression by Statistica software were set at two levels with significance (*p* = 0.05) and remarkable significance (*p* = 0.01).

## 3. Results

### 3.1. Agronomic Characteristics

The results regarding the agronomic characteristics of cannabis plants are presented in Table 3. In both varieties (Fedora 17 and Felina), the presence of *Trichoderma harzianum* (treatments T1 and T2) led to a statistically significant increment of the root density of the plants compared to controls, yet no noteworthy differences were observed between these treatments. The highest root density value was recorded at 2.32 mm cm^−3^ in Fedora 17, where T2 was applied, while the lowest was noted in controls of the same variety (2.02 mm cm^−3^). Treatments T1 and T2 also increased the AMF (Arbuscular Mycorrhizal Fungi) percentage (by 3% and 5.75% in Fedora 17 and by 3% and 6.75% in Felina), though once again no significant differences were noted between the two varieties. The increment of AMF, as expected, led to increased CO_2_ emissions, and especially when the higher dosage of *Trichoderma harzianum* was applied (Table 3). *Trichoderma harzianum* also affected the height of the plants, as well as their dry weight, on both Fedora 17 and Felina. In particular, plant height was increased by 7.5% and 6.6% (on Fedora 17 and Felina, respectively) when 2 × 10^12^ CFU kg^−1^ were applied (T1) and by 12% and 12.5% when 4 × 10^12^ CFU kg^−1^ were applied (T2). Likewise, T1 increased the dry weight of the plants by 10.8% and 9.7%, while T2 increased it by 15.8% and 15% (on Fedora 17 and Felina, respectively). Though T1 and T2 increased both plant height and dry weight, the differences between the two varieties on each treatment were not statistically significant. Finally, regarding the characteristics of the buds, besides their dry weight, where no notable differences were observed, *Trichoderma harzianum* affected their number, their fresh weight, and their moisture. As seen in Table 3, statistically significant differences were reported on the number of buds, as well as their fresh weight, only when the higher dose of *Trichoderma harzianum* was applied. Furthermore, the differences between the two cannabis varieties were found to be insignificant. In contrast to the aforementioned bud characteristics, the moisture of the buds reported a significant increment in response to T1 application (up to 4.25% in Fedora 17 and 6.5% in Felina). It should be noted that on Fedora 17, T2 increased the moisture further by 4.5% (compared to T1).

### 3.2. Yield Characteristics and CBD Content

Regarding the quality characteristics, CBD content and the compact bud index were significantly affected by *Trichoderma harzianum* treatments, as it is shown in Table 4. Furthermore, no interaction between varieties and treatments was mentioned for any yield characteristics. As for dry bud yield, there was no significant difference between the doses of *Trichoderma harzianum* as well as between the two varieties. However, higher values were observed in the Felina variety, and the highest value, 61.70 g plant^−1^, was recorded in Felina in T2. On the other hand, the lowest value, 55.19 g plant^−1^, was presented in Fedora 17 after T2 (Table 4).

The CBD percentage varied among *Trichoderma harzianum* treatments (Table 4). The highest value was 1.32% in Fedora 17 variety after T2, while the lowest was 1.14% in Fedora 17 controls. Moreover, in the Fedora 17 variety, there were no statistically significant differences between control and T1, which were statistically different from T2. As for Felina, there were statistically significant differences between the control and the two different treatments of *Trichoderma harzianum*. CBD yield did not show statistically significant differences between the varieties and the *Trichoderma harzianum* treatments (Table 4). CBD yield ranged from 0.65 g plant^−1^ to 0.80 g plant^−1^ in Fedora 17 controls and Felina in T2, respectively.

The bud length was lowest in Fedora 17 in control 32.25 cm, while the highest value (34.25 cm) was also seen in Felina controls (Table 4). However, the bud length was not affected by the *Trichoderma harzianum* treatments and did not differ between the varieties. The compact bud index was statistically significantly affected by different *Trichoderma harzianum* treatments (Table 4). In Felina, the values ranged from 3.35 fw/cm gr/cm (control) to 3.91 fw/cm gr/cm (T2). Moreover, in Fedora 17, there were no significant differences between the control and the T1, which differed significantly from T2.

## 4. Discussion

The root density of *Cannabis sativa* plants seems to increase through the application of AMF (r = 0.67; *p* < 0.001) (Table 5). The presence of *Trichoderma harzianum* has a positive effect on the root system of many plants [51,52]. A strong positive correlation was also noted between AMF and plant characteristics such as plant height (r = 0.84; *p* < 0.001) and plant above-ground dry weight (r = 0.81; *p* < 0.001). Various studies on cucumber, lettuce, and cabbage have reported similar results [51,53]. Plant growth promotion is caused by mycorrhizae fungi through many mechanisms such as competing pathogens, hormone production, and mainly due to an increase in mineral uptake from the soil [54]. The last one is considered as the main factor since nutrients uptake was noticed even in sterile soils in the absence of pathogens [55,56,57]. Thus, the abilities of *Trichoderma harzianum* to solubilize minerals such as Fe, Mn, Zn, P [58], and offer additional N to the plant [59], and the fact that its presence shortens the distance that nutrients must diffuse towards the roots [55], could be responsible for the increment of root density, height and dry matter of hemp plants, as well as other agronomic characteristics such as the number of buds per plant and their fresh weight [55,58,59].

Furthermore, research published by Papastylianou et al. in 2018 [2] indicated that the application of N fertilization positively impacted inflorescence indices in hemp crops. Thus, the potential enhancement in N uptake caused by AMF could lead to an increase in bud numbers and their weight. This hypothesis is compatible with the results of this present study as parallelisms between AMF levels, the number of buds per plant (r = 0.56; *p* < 0.01), and fresh weight of buds (r = 0.64; *p* < 0.001) were noted. A close correlation between root density, number of buds per plant (r = 0.81; *p* < 0.001) and bud fresh weight (r = 0.68; *p* < 0.001) was also reported, as seen in Table 5. As expected, an increased number of buds would lead to increased bud dry yields (r = 0.65; *p* < 0.001), whilst greater root densities would increase the overall water uptake of the plants and therefore improve the average moisture of the buds (r = 0.63; *p* < 0.001). Having taken into account the fact that the compact bud index is the ratio of their fresh weight and their length, the positive correlation between the index and the fresh weight of the buds (r = 0.78; *p* < 0.001) and the negative one between the index and the average bud length (r = –0.57; *p* < 0.01), found in the present study, were anticipated.

CBD concentrations in the buds were also affected by the application of AMF (r = 0.66; *p* < 0.001 and r = 0.50; *p* < 0.05, respectively) (Table 5). As mentioned before, *Trichoderma harzianum* is capable of providing additional P to the plants. Generally, it is believed that minerals heavily affect the synthesis of secondary metabolites in plants [59]. Particularly, P has been found to affect the production of CBD in cannabis plants [60]. Therefore, CBD concentrations may have been raised due to P accumulation in the roots provided by AMF. This would also explain the close correlation between CBD percentages and the root density (r = 0.84; *p* < 0.001) that can be seen in Table 5.

It should be mentioned that in 2019, Bernstein et al. claimed that treating hemp plants with P does not affect the concentration of CBD [14]. Notably, though, the CBD yield reported had the closest correlation with the dry yield of the buds (r = 0.93; *p* < 0.001).

Fungi that belong to *Trichoderma* genus are previously reported to promote root growth [61] and enhance internal biochemical changes in plant tissues through the release and reciprocity of various secondary metabolites after root colonization [34,62]. Harzianic acid (HA) is among the metabolites of *Trichoderma harzianum*, responsible for plant growth promotion [63]. Their application is not limited only to plants’ rhizosphere but also to aboveground biomass. The foliar treatment of *Passiflora caerulea* with *Trichoderma* spores promoted the photosynthetic capacity of the species, which, in turn, entrained the yield enhancement [64]. Regarding soil activity of the fungus, the association with soil minerals and the solubilization of phosphate is a well-known process that is carried out by *Trichoderma* fungi and highlights the promotion of mobility of this important macronutrient for plants [65]. The moderation of nitrogen and carbon concentration in the soil and the plant, along with Fe^3+^ chelating siderophores production, is a process that leads to increased nutrient availability [66]. However, there are also reports about potential negative effects on the crop due to the production of phytotoxic metabolites (trichothecenes) [67].

The inoculation of *Trichoderma* spp. in plants might be beneficial under salt stress conditions by promoting biomass accumulation and improving water use efficiency, as reported previously in *Triticum aestivum* [68]. Beyond physiological traits amplification, specific morphological alterations have also been observed in soybean treated with *Trichoderma harzianum*, where stomatal density was increased [69]. *Trichoderma* shows high adaptability potential in various soil conditions, while pH has been reported as a factor that strongly affected the *Trichoderma harzianum* T059 strain development [70]. Another *Trichoderma harzianum* strain (Tr904) has been isolated from the wheat rhizosphere at 0–10 cm soil depth, where is persistently associated with the root system of the crop [71]. Isolation from greater depth was decreased, indicating that *Trichoderma*’s active growth zone is near the ground surface. In the current study, T-22 strain spores were used to promote the growth of hemp plants through continuous fertigation 10 and 30 days after sowing. The increase in the *Trichoderma harzianum* dose to 4 × 10^12^ cfu kg^−1^ led to a significant improvement in all examined characteristics, acting as a biostimulant [64,68].

The initial hypothesis that an increased rate of *Trichoderma harzianum* with only two applications could sufficiently colonize roots and promote the mobilization of nutrients in the aboveground plant tissues was confirmed. The interpretation of this result might be explained due to the initial dense colonization of cannabis rhizosphere by *Trichoderma* conidia and moderation of carbon allocation, along with the secretion of indole-3-acetic-acid (IAA) and other secondary metabolites [72] that guide plant growth, altering the morphological traits of cannabis. Phytohormones (including auxins and gibberellins) production fortification after the colonization process could explain the significant increment of important agronomic traits of hemp, such as plant height and root density [73].

The significance of *Trichoderma harzianum* is not only hidden in the promotion of plant development but also in several processes, for instance, the chemical composition of plant material. Considering the accumulation of secondary metabolites, the literature recognizes the impact of environmental conditions such as light, water availability, and temperature on the synthesis of those compounds [59]. Among them, nutrient availability is mentioned as one of the most determinant factors for the production of secondary metabolites. In the case of hemp, nitrogen supply positively affects the secondary metabolites, especially cannabinoids [74]. Previous studies reveal that the addition of NPK fertilizer raised the concentration of cannabinol (CBN), while CBN content augmented after the supply of organic fertilizer [75].

*Trichoderma harzianum* meets this need for nutrient availability as it solubilizes minerals such as Fe, Mn, Zn, P resulting in the supply of additional N to the plant [58]. As a result, it is expected an increase in secondary metabolites after the colonization of *Trichoderma harzianum*. This hypothesis is well depicted in our experiment, and marked differences were observed in CBD content between control plants and inoculated with *Trichoderma harzianum* hemp plants. We propose that the root colonization by *Trichoderma harzianum* entrains a sequence of biochemical processes which amplify the polyketide and the plastidial DOXP/MEP pathways for cannabinoid production such as THC and CBD, which has been recently presented by Lowe et al. [76]. This hypothesis is underpinned by the well-known production of multiple volatile organic compounds by *Trichoderma* species that promote the plant growth of plants [77] and the secretion of small molecules, such as polyketides, observed in *Trichoderma harzianum* T-22 [78]. Moreover, a second indirect pathway that might be beneficial for CBD content increase is the enhancement of phosphorus availability [79]. *Trichoderma* strains phosphate solubilization activity has been previously reported [80]. However, specific contributions of the fungus to precursor molecules of CBD had not been examined because it was outside of the scope of this study. Both monoecious varieties (Fedora 17 and Felina) were affected by the inoculation with almost similar CBD content. It is also worth mentioning that CBD content had a strong correlation with the number of inflorescences per plant (r = 0.84, *p* < 0.01) (Table 5).

To the best of our knowledge, this is the first report of *Trichoderma harzianum* application to a trade product in a species of *Cannabaceae* family that thoroughly examines the impact of increased doses of fungi supply in the agronomic and quality characteristics of *Cannabis sativa*. Crop production is mainly linked with end-products quality, nutritional and economic value. Hemp is cultivated both in greenhouses and in the field purposely for its cannabidiol (CBD) [81]. Hence, it remains crucial to assist secondary metabolites production and promote the balanced mobilization of nutrients in root, stem, and leaves. *Trichoderma harzianum* strain T22 was revealed in our study as a potential biofertilizer for *C. sativa* to moderate CBD content. This outcome is consistent with the findings of Carillo et al. [82], who confirmed that the addition of T22 strain provoked the increase in lycopene in *Solanum lycopersicum*, a desirable antioxidant compound, and Pascale et al. [83], who observed the yield and antioxidant capacity enhancement of grapes.

Further extensive research should be conducted to understand better the implications of *Trichoderma*-cannabis associations. The interaction of the fungus with roots is a complex framework that includes several secondary metabolites secretion, rapid metabolomic alterations, and biochemical pathway triggers. *Trichoderma harzianum* utilization could be extended beyond its typical use as a biological agent of pesticides against several plant diseases [84,85] and be integrated as a component of integrated fertilization programs [86] for sustainable crop production of major field and industrial crops, such as hemp. This study reveals that *Trichoderma harzianum* can significantly improve hemp production without the combined application with AMF [46,87].

## 5. Conclusions

**Figure 1:**
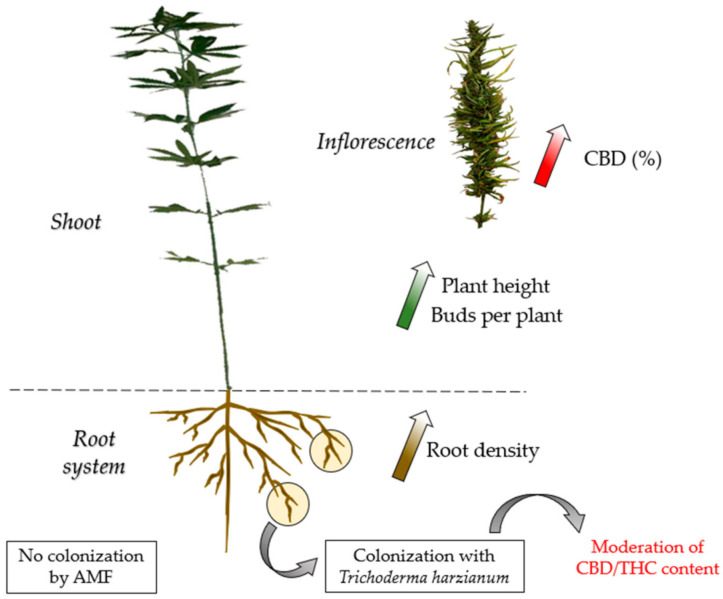
Positive impact of cannabis colonization with *Trichoderma harzianum* on three axes: root structure, vigor, CBD content.

In conclusion, utilization of *Trichoderma harzianum* as a PGPM seems to boost the growth of *Cannabis sativa* plants and increase the content of CBD (Figure 1). However, CBD yield was not affected by its presence. Furthermore, following its application, significant differences were reported in the majority of the agronomic characteristics of the plants. In particular, root density, plant height, aboveground dry weight, and the number of buds per plant were significantly increased (Figure 1). Moreover, T1 and T2 treatments did not present notable differences, thus indicating that lower doses (2 × 10^12^ CFU kg^−1^) of *Trichoderma* are sufficient. CBD content was maximized when higher doses were applied (4 × 10^12^ CFU kg^−1^). Although the results of this present study suggest a high potentiality in the use of *Trichoderma harzianum* as a plant-growth promotion microorganism, further research should be conducted regarding the mechanism (or mechanisms) behind its beneficial effects on *Cannabis sativa*.

## Figures and Tables

**Table 1:** Soil and compost physicochemical properties.

	pH	Olsen-P (mg kg^−1^ Soil)	Available Potassium (K) (mg kg^−1^ Soil)	Organic Matter (%)
Soil	7.36	15	215	1.36
Compost	7.6	410	620	42.41

**Table 2:** Treatments and total *Trichoderma harzianum* applied rates.

Treatment	*Trichoderma harzianum* Amount Added per Pot (g)	Total Applied Fungus Spores *CFU* kg^−1^
Control	−	−
T1	2	2 × 10^12^
T2	4	4 × 10^12^

**Table 3:** Agronomic characteristics as affected by different treatments of *Trichoderma harzianum* in hemp varieties, Fedora 17 and Felina.

	Root Density (mm cm^−3^)		AMF (%)		CO_2_ mg/100 g/24 h/25 °C		Plant Height (cm)		DW/Plant (g)		Number of Buds per Plant		Bud Weight Fresh (g)		Bud Moisture (%)		Bud Weight Dry (g)	
	Fedora 17	Felina	Fedora 17	Felina	Fedora 17	Felina	Fedora 17	Felina	Fedora 17	Felina	Fedora 17	Felina	Fedora 17	Felina	Fedora 17	Felina	Fedora 17	Felina
**Control**	1.99 ^a^	2.02 ^a^	19.75 ^a^	19.75 ^a^	51.50 ^a^	50.25 ^a^	126.75 ^a^	127.50 ^a^	199.75 ^a^	201.75 ^a^	1.12 ^a^	1.16 ^a^	113.75 ^a^	114.75 ^a^	56.25 ^a^	54.50 ^a^	49.79 ^ns^	52.28 ^ns^
**T1**	2.25 ^b^	2.25 ^b^	22.75 ^b^	22.75 ^b^	58.50 ^ab^	60.25 ^b^	136.25 ^b^	136 ^b^	221.25 ^b^	221.25 ^b^	1.19 ^ab^	1.21 ^ab^	117.75 ^a^	118 ^a^	60.50 ^b^	61 ^b^	46.59 ^ns^	46.09 ^ns^
**T2**	2.32 ^b^	2.30 ^b^	25.50 ^c^	26.50 ^c^	64.50 ^b^	67.75 ^b^	142 ^b^	143.50 ^b^	231.25 ^b^	232 ^b^	1.26 ^b^	1.27 ^b^	124.50 ^b^	131 ^b^	65 ^c^	63 ^b^	43.64 ^ns^	48.44 ^ns^
**F_Doses_**	23.39 ***		34.32 ***		25.23 ***		22.62 ***		38.75 ***		8.78 **		7.58 **		34.70 ***		ns	
**F_Variety_**	ns		ns		ns		ns		ns		ns		ns		ns		ns	
**F_Doses × Variety_**	ns		ns		ns		ns		ns		ns		ns		ns		ns	

F-test ratios from ANOVA. Different letters (^a^, ^b^, and ^c^) within a column indicate significant differences according to Tukey’s test. Significance levels: ** *p* < 0.01; *** *p* < 0.001; ns, not significant (*p* > 0.05); AMF: Arbuscular Mycorrhizal Fungi.

**Table 4:** Yield characteristics and CBD content as affected by different *Trichoderma harzianum* treatments in hemp varieties Fedora 17 and Felina.

	Bud Dry Yield(g plant^−1^)		Bud Length(cm)		Bud Compact Index(fw/cm gr/cm)		CBD(%)		CBD Yield(g plant^−1^)	
	Fedora 17	Felina	Fedora 17	Felina	Fedora 17	Felina	Fedora 17	Felina	Fedora 17	Felina
**Control**	56.14 ^ns^	60.65 ^ns^	32.25 ^ns^	34.25 ^ns^	3.54 ^a^	3.35 ^a^	1.14 ^a^	1.18 ^a^	0.65 ^ns^	0.72 ^ns^
**T1**	55.66 ^ns^	55.91 ^ns^	33.75 ^ns^	32.75 ^ns^	3.49 ^a^	3.63 ^b^	1.23 ^a^	1.24 ^b^	0.69 ^ns^	0.70 ^ns^
**T2**	55.19 ^ns^	61.70 ^ns^	33 ^ns^	33.50 ^ns^	3.78 ^b^	3.91 ^c^	1.32 ^b^	1.29 ^b^	0.73 ^ns^	0.80 ^ns^
**F_Doses_**	ns		ns		3.90 *		7.27 **		ns	
**F_Variety_**	ns		ns		ns		ns		ns	
**F_Doses × Variety_**	ns		ns		ns		ns		ns	

F-test ratios for dose, variety and their combination are from ANOVA. Different letters (^a^, ^b^ and ^c^) within a column indicate significant differences according to Tukey’s test. Significance levels: * *p* < 0.01; ** *p* < 0.01; ns, not significant (*p* > 0.05); CBD: cannabidiol.

**Table 5:** Correlation matrix between agronomic, yield characteristics, and CBD content of hemp varieties (Fedora 17 and Felina).

	Root Density (mm cm^−3^)	AMF(%)	CO_2_ mg/100 g/24 h/25 °C	Plant Height (cm)	DW/Plant (g)	Number of Buds per Plant	Bud Weight Fresh (g)	Bud Moisture (%)	Bud Weight Dry (g)	Bud Dry Yield(g plant^−1^)	CBD(%)	CBD Yield(g plant^−1^)	Bud Length (cm)	Bud Compact Index (fw/cm gr/cm)
**Root density (mm cm^−3^)**	1.00	0.67 ***	0.60 **	0.76 ***	0.94 ***	0.81 ***	0.68 ***	0.63 ***	−0.14 ^ns^	0.28 ^ns^	0.84 ***	0.56 **	0.18 ^ns^	0.46 *
**AMF (%)**		1.00	0.70 ***	0.84 ***	0.81 ***	0.56 **	0.64 ***	0.81 ***	−0.37 ^ns^	−0.02 ^ns^	0.50 *	0.20 ^ns^	−0.07 ^ns^	0.57 **
**CO_2_ mg/100 g/24 h/25 °C**			1.00	0.59 **	0.72 ***	0.52 **	0.38 ^ns^	0.77 ***	−0.51 *	−0.15 ^ns^	0.41 *	0.06 ^ns^	−0.21 ^ns^	0.44 *
**Plant height (cm)**				1.00	0.81 ***	0.71 ***	0.71 ***	0.71 ***	−0.22 ^ns^	0.17 ^ns^	0.61 **	0.38 ^ns^	0.09 ^ns^	0.53 **
**DW/plant (g)**					1.00	0.73 ***	0.65 ***	0.73 ***	−0.27 ^ns^	0.14 ^ns^	0.78 ***	0.43 *	0.12 ^ns^	0.47 *
**No buds per plant**						1.00	0.82 ***	0.38 ^ns^	0.22 ^ns^	0.65 ***	0.84 ***	0.83 ***	−0.02 ^ns^	0.70 ***
**Bud weight fresh (g)**							1.00	0.37 ^ns^	0.35 ^ns^	0.66 ***	0.69 ***	0.77 ***	0.06 ^ns^	0.78 ***
**Bud moisture (%)**								1.00	−0.74 ***	−0.40 ^ns^	0.44 *	−0.12 ^ns^	−0.12 ^ns^	0.36 ^ns^
**Bud weight Dry (g)**									1.00	0.88 ***	0.06 ^ns^	0.68 ***	0.16 ^ns^	0.20 ^ns^
**Bud Dry yield(g plant^−1^)**										1.00	0.45 *	0.93 ***	0.11 ^ns^	0.49 *
**CBD (%)**											1.00	0.74 ***	0.14 ^ns^	0.48 *
**CBD yield (g plant^−1^)**												1.00	0.15 ^ns^	0.56 **
**Bud length (cm)**													1.00	−0.57 **
**Bud Compact index(fw/cm gr/cm)**														1.00

Significance levels: * *p* < 0.05; ** *p* < 0.01; *** *p* < 0.001; ns, not significant (*p* > 0.05).

## Data Availability

The data presented in this study are available on request from the corresponding author.

## Abbreviations

AMF: arbuscular mycorrhiza fungi; CBD, cannabidiol; THC, Δ9-tetrhydrocannabinol; N, nitrogen; PGPM, plant growth-promoting microorganisms; CFU, colony forming units; DAS, days after sowing; FAA, formalin/acetic acid/alcohol; DW, dry weight; P, phosphorus; IAA, indole-3-acetic-acid; CBN, cannabinol.
